# Maternal Glucose Metabolism and Emotional and Behavioral Problems in Offspring: Modification by Erythrocyte Polyunsaturated Fatty Acids

**DOI:** 10.3390/nu18121840

**Published:** 2026-06-06

**Authors:** Xuanqing He, Sufang Duan, Jian He, Bin Sun, Ting Li, Minyan Lan, Xiaonan Gu, Guoyu Zhang, Lizi Lin, Duo Li, Li Cai

**Affiliations:** 1 Department of Maternal and Child Health, School of Public Health, Sun Yat-sen University, Guangzhou 510080, China; hexq29@mail2.sysu.edu.cn (X.H.);; 2 National Technology Innovation Center for Dairy, Hohhot 010110, China; 3 Yili Maternal and Infant Nutrition Institute (YMINI), Inner Mongolia Yili Industrial Group, Co., Ltd., Beijing 100071, China; 4 Joint International Research Laboratory of Environment and Health, Ministry of Education, Guangdong Provincial Engineering Technology Research Center of Environmental Pollution and Health Risk Assessment, Sun Yat-sen University, Guangzhou 510080, China; 5 Institute of Nutrition & Health, Qingdao University, Qingdao 266071, China

**Keywords:** glucose metabolism, emotional and behavioral problems, polyunsaturated fatty acids, pregnancy, children

## Abstract

**Background:** Gestational diabetes mellitus (GDM) adversely affects offspring neurobehavioral outcomes, yet evidence regarding continuous markers of maternal glucose metabolism remains limited. Although polyunsaturated fatty acids (PUFAs) have been shown to affect associations between glucose metabolism and respiratory outcomes, their effects on children’s emotional and behavioral problems remain unclear. This study investigated the association between maternal glucose metabolism and emotional and behavioral problems in children and the potential modifying effect of maternal erythrocyte PUFAs. **Methods:** This prospective birth cohort included 481 mother–child pairs. Maternal glucose metabolism during pregnancy was assessed using GDM diagnosis via a 75 g oral glucose tolerance test (OGTT), fasting plasma glucose (FPG), 1 h and 2 h OGTT glucose, hemoglobin A1c (HbA1c), insulin, and insulin resistance (HOMA-IR). Maternal erythrocyte PUFAs were quantified by gas chromatography. Children’s emotional and behavioral problems at age 5 years were assessed using the Strengths and Difficulties Questionnaire (SDQ). Generalized linear models were used to evaluate associations, including multiplicative interaction terms between glucose metabolism indicators and PUFAs. **Results:** Maternal FPG (OR = 1.63; 95%CI: 1.08–2.47), OGTT-1h glucose (OR = 1.84; 95%CI: 1.08–3.12), and HOMA-IR (OR = 1.52; 95%CI: 1.01–2.27) were each positively associated with an increased risk of abnormal total difficulties scores in children. Maternal insulin levels were positively associated with abnormal total difficulties scores in girls (*p* for interaction < 0.05). Higher maternal *n*-3 PUFA levels and lower *n*-6 PUFA levels attenuated the risk of glucose metabolism-related emotional and behavioral problems in children. **Conclusion:** Maternal glucose metabolism was associated with increased risk of emotional and behavioral problems in children. PUFA biomarkers could modify glucose-related emotional and behavioral outcomes in children.

## 1. Introduction

Emotional and behavioral problems are common mental health issues in children and are frequently observed in the preschool period [1]. Globally, approximately 13.4% of children and adolescents suffer from mental disorders. In China, the prevalence of emotional and behavioral problems was 11.2% among preschool children, posing a significant threat to their learning and physical and mental health [2,3]. The occurrence of these problems is associated with brain regions such as the prefrontal cortex, which are vulnerable to adverse stimuli during pregnancy and the perinatal period [4]. Notably, abnormal glucose metabolism, a common complication during pregnancy, may impair early fetal neurodevelopment [5].

Gestational diabetes mellitus (GDM) is the most prevalent metabolic disorder during pregnancy, with a global prevalence of approximately 14% that continues to rise [6]. Notably, the prevalence in China has reached 15.7% [7]. Some studies in European and American populations have reported a positive association between maternal GDM and offspring emotional and behavioral problems [8,9,10], which appears to be sex-specific. A Finnish study found that boys born to GDM mothers were more likely to exhibit hyperactivity/inattention [11], while another study suggested that girls showed more pronounced hyperglycemia-related behavioral disorders [12]. Notably, few studies have focused on East Asian populations, who exhibit a higher susceptibility to GDM. Furthermore, existing research has demonstrated a linear association between continuous glycemic indicators and adverse pregnancy outcomes, with each indicator reflecting distinct aspects of the metabolic physiological state [13]. Given the heterogeneity of glucose metabolism markers, different GDM phenotypes may exert varying effects on offspring health outcomes [14,15,16]. However, the associations between these continuous glycemic indicators and offspring emotional and behavioral problems remain insufficiently elucidated.

Maternal nutritional factors during pregnancy are also critical determinants of fetal neuropsychological development. Polyunsaturated fatty acids (PUFAs) have garnered significant attention due to their antioxidant and anti-inflammatory properties [17]. They play an important role in maintaining central nervous system integrity by regulating neuronal membrane fluidity and modulating neuroinflammation [18]. Multiple studies have confirmed that PUFAs can improve insulin sensitivity in GDM and are involved in neural signaling during fetal brain development [19,20]. Although some studies indicate that PUFAs may improve offspring health outcomes related to maternal glycemic levels during pregnancy [21,22], no evidence exists in the domain of emotional and behavioral problems, particularly in the context of transgenerational research.

This birth cohort study investigated the association between maternal glucose metabolism during pregnancy and offspring emotional and behavioral problems at five years of age. We further explored whether maternal PUFAs, measured using erythrocyte biomarkers, modified this association.

## 2. Methods

### 2.1. Study Population

**Figure 1:**
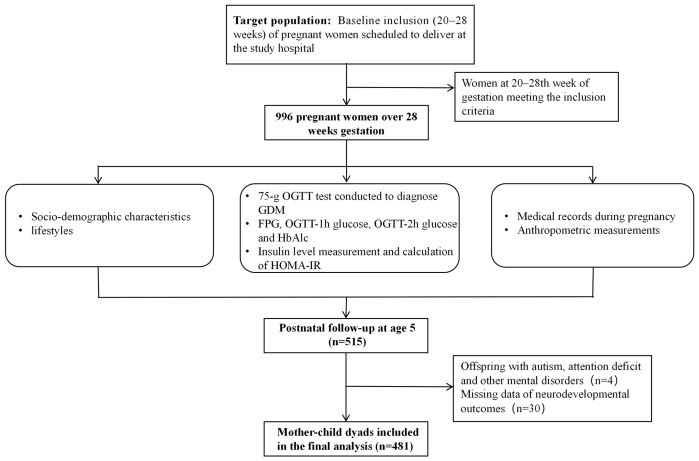
The diagram depicts the flow chart of the inclusion crowd.

This prospective cohort study was conducted from March 2017 to November 2018 at Yuexiu District Maternal and Child Health Hospital in Guangzhou, China. Eligible participants were singleton mothers aged 20–45 years, without a history of gestational diabetes, cardiovascular diseases, thyroid diseases, blood diseases, polycystic ovary syndrome, infectious diseases, or mental illnesses, and with available data on maternal mid-pregnancy blood glucose levels. A total of 996 mother–child pairs were included. In this study, parents of 515 children completed follow-up assessments of emotional and behavioral problems at the age of 5. After further excluding participants with a diagnosed mental illness in children and missing data on emotional and behavioral problems (*n* = 34), the final analytic sample comprised 481 mother–child dyads. The included population had higher educational levels compared to the non-included population (Table S1). The study population inclusion flow chart is shown in Figure 1. This study was approved by the Ethics Committee of Sun Yat-sen University (Approval No.: SYSU-PHME [2016] No. 014, Date: 28 December 2016), and all participants provided written informed consent.

### 2.2. Measurement of Glucose Metabolism in the Second Trimester

Between 20 and 28 weeks of gestation, pregnant women underwent a standard 75 g oral glucose tolerance test (OGTT) after a ≥10 h fast, administered by trained clinical nurses. Plasma glucose levels were measured using the glucose oxidase method (ARCHITECT i2000SR; Abbott Laboratories, Abbott Park, IL, USA). The glucose measurements included fasting plasma glucose (FPG), OGTT after one hour (OGTT-1h) glucose, OGTT after two hours (OGTT-2h) glucose, and hemoglobin A1c (HbA1c). Insulin indicators were detected by Enzyme-Linked Immunosorbent Assay (ELISA). GDM was diagnosed according to the criteria of the International Association of the Diabetes and Pregnancy Study Groups (IADPSG), defined as meeting any of the following thresholds: FPG ≥ 5.10 mmol/L, OGTT-1h glucose ≥ 10.00 mmol/L, or OGTT-2h glucose ≥ 8.50 mmol/L [23]. Insulin resistance was evaluated using the homeostasis model assessment of insulin resistance (HOMA-IR), calculated as HOMA-IR = (FPG × fasting insulin)/22.5 [24].

### 2.3. Assessment of Emotional–Behavioral Problems in Children at Age 5

Children’s emotional and behavioral problems were assessed at age 5 through parent–clinician interviews using the Strengths and Difficulties Questionnaire (SDQ), which has been verified to be reliable and effective in the Chinese population [25]. This 25-item scale comprises five domains: emotional symptoms, conduct problems, hyperactivity/inattention, peer relationship problems, and prosocial behavior, each scored from 0 to 10. The total difficulties score is calculated as the sum of the first four domains. A higher total difficulties score indicates more severe emotional and behavioral problems. Conversely, a higher score in prosocial behavior reflects stronger social abilities. According to previous studies, “borderline” and “abnormal” scores were combined and categorized as “abnormal” for analysis to increase statistical power [26]. The total difficulties score was treated as the primary outcome, while domain-specific analyses were conducted as secondary outcomes [27].

### 2.4. Analysis of PUFA Biomarkers

Maternal blood samples were collected after a ≥10 h fast between 20 and 28 weeks of gestation. Following the method described by Peng et al. [28], fatty acid methyl esters (FAMEs) were extracted via transesterification with 5% (*v*/*v*) sulfuric acid in methanol and toluene at 70 °C for 2 h. Samples were analyzed using an Agilent 7820 gas chromatograph (Agilent Technologies, Santa Clara, CA, USA) with a flame ionization detector and a DB-23 capillary column (60 m × 0.25 mm ID × 0.25 μm film thickness). Nitrogen served as the carrier gas at 300 kPa. Injector and detector temperatures were set to 270 °C. The program heating lasted 13 min, with the oven temperature heating from 150 °C to 230 °C. PUFAs were identified using FAME standards (Nu-Chek Prep, Inc, Waterville, MN, USA) and quantified with an internal standard (1 mg per 500 mg sample). Fatty acid levels were expressed as percentages of total peak area, and the *n*-6/*n*-3 ratio was calculated by dividing total *n*-6 PUFAs (%) by total *n*-3 PUFAs (%). PUFA levels were analyzed as continuous variables.

### 2.5. Covariates

Sociodemographic data were collected during baseline interviews, including maternal age, educational level (<high school, high school to junior college, ≥University), smoking status (including passive smoking), and family history of diabetes. Pre-pregnancy body mass index (BMI, kg/m^2^) was calculated from measured height (nearest 0.1 cm) and self-reported pre-pregnancy weight, while gestational weight gain was the difference between pre-pregnancy weight and weight at the last prenatal visit. Clinical data such as gestational age at delivery, mode of delivery, infant sex, and birth weight were obtained from hospital records.

Data on breastfeeding duration (categorized as <4 months, 4 to <6 months, or ≥6 months) and the age of complementary food introduction (<6 months or ≥6 months) were collected through one-on-one interviews at 6 months of age.

### 2.6. Statistical Analysis

All analyses were performed using R software version 4.4.0 (R Core Team, Vienna, Austria). Continuous variables are presented as means ± standard deviations (SD), and categorical variables as frequencies and percentages. The normality of continuous variables was assessed using the Shapiro–Wilk test. Differences between the GDM and non-GDM groups were evaluated using independent *t* tests for normally distributed continuous variables, Wilcoxon rank-sum tests for non-normally distributed continuous variables, and chi-square tests for categorical variables.

Associations between maternal glucose metabolism during pregnancy and emotional–behavioral problems in offspring at age 5 were analyzed using multiple logistic regression models. The main models adjusted for potential confounders, including maternal age, educational level, pre-pregnancy BMI, gestational weight gain, family history of diabetes, smoking status, physical activity, dietary energy intake, infant sex, mode of delivery, gestational age at delivery, birth weight, duration of exclusive breastfeeding, and age at introduction of complementary foods. A restricted cubic spline function was used to explore nonlinear dose–response relationships. Exploratory stratified analyses were conducted on the total difficulties score based on child sex.

Interaction analyses were performed by including cross-product terms between continuous maternal glucose metabolism indicators and erythrocyte PUFA levels in the regression models. To visualize the direction and magnitude of the interaction effects, we stratified PUFAs at the 10th, 50th, and 90th percentiles using the ‘visreg’ package to illustrate interaction patterns.

Sensitivity analyses included (a) excluding infants born prematurely (<37 weeks) or with abnormal birth weight (<2500 g or >4000 g); (b) adjusting for illness and outdoor activities in children.

## 3. Results

### 3.1. Characteristics of the Study Population

Table 1 summarizes the demographics of the 481 mother–child pairs. A total of 19.33% of the subjects were diagnosed with GDM. Women with GDM were older and had higher pre-pregnancy BMI than those without GDM (*p* < 0.05). Conversely, non-GDM mothers were more likely to have vaginal deliveries and greater gestational weight gain (*p* < 0.05). Approximately half of the children were male (48.0%). The prevalence of abnormal total difficulties scores among children was 13.31%.

### 3.2. Maternal Blood Glucose Metabolism Levels and Distribution of PUFAs

In the general population, the abnormal rates of FPG, OGTT-1h and OGTT-2h glucose were 4.99%, 9.77% and 11.85% respectively. Compared with the non-GDM group, glucose metabolism levels were significantly higher in the GDM group (*p* < 0.05; Table 2). The mean maternal levels of *n*-3 PUFAs, *n*-6 PUFAs, and the *n*-6/*n*-3 PUFA ratio were 9.70%, 35.04%, and 3.91, respectively, with no significant differences observed between the GDM and non-GDM groups.

### 3.3. Results of Children’s Emotional–Behavioral Problems

**Figure 2:**
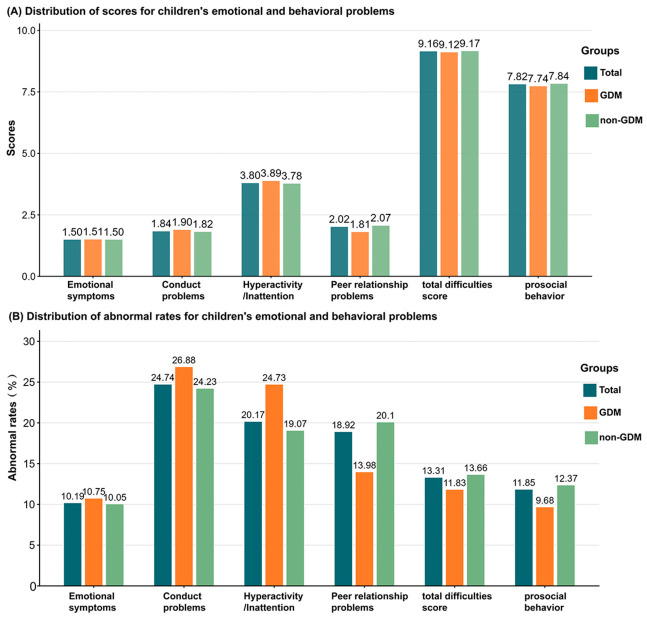
SDQ scores and abnormalities in offspring at 5 years of age. SDQ, the Strengths and Difficulties Questionnaire; GDM, gestational diabetes mellitus; SD, standard deviation.

As shown in Figure 2, the abnormal rate of emotional problems in children in the total population was the lowest (10.19%), followed by prosocial behavior problems (11.85%), peer relationship problems (18.92%), hyperactivity/inattention (20.17%), and conduct problems (24.74%). A total of 52.6% of children had abnormal scores in at least one domain.

### 3.4. Relationship Between Glucose Metabolism Level During Pregnancy and Emotional and Behavioral Problems in Offspring at Age 5

In the main model, per 1-SD increase in maternal FPG, OGTT-1h glucose, and HOMA-IR was associated with an elevated risk of abnormal total difficulties scores in children, with odds ratios (ORs) and 95% confidence intervals (CIs) of 1.58 (1.07, 2.32), 1.58 (1.06, 2.35), and 1.67 (1.12, 2.50), respectively. GDM was positively associated with the risk of abnormal hyperactivity/inattention (OR, 2.05; 95% CI: 1.04–4.04), HbA1c was positively associated with the risk of abnormal conduct problems (OR, 1.38; 95% CI: 1.01–1.88) (Table 3). No significant nonlinear associations were observed. (Figure S1).

### 3.5. Modification Effects of PUFAs

**Figure 3:**
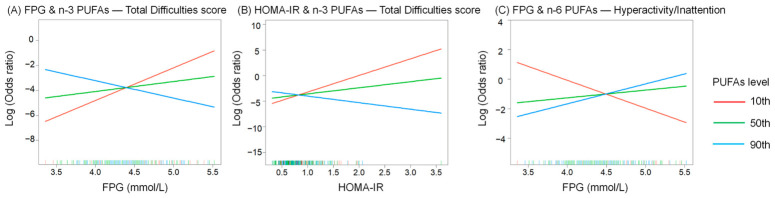
Interaction between maternal erythrocyte PUFAs and glucose metabolism levels. The vertical coordinate represents the OR value of the interaction between glucose metabolism index and fatty acid. The horizontal coordinate represents the glucose metabolism index. The model was adjusted for maternal age, maternal educational level, pregnancy BMI, gestational weight gain, family history of diabetes, maternal smoking, physical activity, dietary energy intake, children sex, mode of delivery, weeks of gestation, birth weight, exclusive breastfeeding and complementary feeding age. OR, odds ratio; PUFAs, polyunsaturated fatty acids; OGTT, oral glucose tolerance test; HbA1C, glycosylated hemoglobin; HOMA-IR, HOMA-Insulin Resistance.

The interaction diagrams are shown in Figure 3. Higher erythrocyte *n*-3 PUFA levels were associated with attenuated adverse relationships between FPG and total difficulties score (OR_interaction_ = 0.52; 95% CI: 0.30–0.91), as well as between HOMA-IR and total difficulties score (OR_interaction_ = 0.51; 95% CI: 0.29–0.91). In contrast, higher erythrocyte *n*-6 PUFA levels were associated with a strengthened adverse relationship between FPG and hyperactivity/inattention (OR_interaction_ = 1.63; 95% CI: 1.05–2.51) (Table S2).

### 3.6. Sensitivity Analysis

After excluding premature births, low birth weights, and macrosomia infants (Tables S3 and S4) and adjusting for sensitivity analysis of disease and outdoor activities in children (Tables S5 and S6), the association between glucose metabolism during pregnancy and emotional and behavioral problems in offspring remained largely consistent with what was observed in the main model.

### 3.7. Stratified Analysis

Maternal insulin levels were positively associated with abnormal total difficulty scores among girls (OR = 2.87; 95% CI: 1.37–5.99) and the difference between strata was statistically significant (*p* for interaction < 0.05) (Table S7).

## 4. Discussion

In this prospective birth cohort study conducted in China, the study found that measures of glucose metabolism during pregnancy were associated with an increased risk of emotional and behavioral problems in children at the age of 5, with the associations related to insulin being more pronounced in girls. For the first time, the present study demonstrates that this association is strengthened when the levels of *n*-3 PUFAs are lower and attenuated when *n*-6 PUFAs are higher.

The findings of this study regarding the association between maternal GDM and offspring emotional and behavioral problems are supported by four prospective cohort studies from North America [8,9,10,29]. These studies consistently reported associations between GDM and greater emotional and behavioral difficulties in offspring across multiple assessment tools and ages ranging from 2 to 10 years. In contrast, cohort studies from Greece and Australia did not observe a significant association between GDM and childhood emotional and behavioral outcomes, which may be attributed to differences in assessment tools and age groups [30,31]. Furthermore, the results of this study concerning the association with continuous glycemic metabolism indicators show that FPG and OGTT-1h levels were positively correlated with offspring emotional and behavioral problems, consistent with two previous studies [8,32]. The discrepancy with the Greek study may be due to differences in the timing of blood glucose measurements [31]. However, mid-pregnancy blood glucose levels are less susceptible to transient factors such as diet and are relatively stable and reliable. Notably, our study is the first to demonstrate positive associations between HOMA-IR, HbA1c, and emotional and behavioral problems in offspring. HOMA-IR can more sensitively capture the early decline in insulin sensitivity, reflecting glucose metabolism abnormalities earlier and more comprehensively [33]. HbA1c reflects the average blood glucose level over the past 8 to 12 weeks and can serve as an indicator of potential glucose metabolism abnormalities in pregnant women who have not yet exhibited obvious hyperglycemic symptoms [34]. Therefore, while continuing to emphasize early screening and treatment for GDM, future efforts should also strengthen the management of individuals who do not meet the GDM diagnostic criteria but exhibit other indicators of glucose metabolism abnormalities, thereby effectively improving maternal and child health outcomes.

Further stratified analysis in this study found that the association between maternal insulin levels and abnormal total difficulties scores in offspring was more pronounced in female offspring. Previous studies have often reported more severe emotional and behavioral problems related to glucose metabolism disorders in male children [11,35]; however, our findings suggest that insulin-related pathways may play a key role in female children. This may be due to excessively high insulin levels during pregnancy affecting brain-derived Neurotrophic factor expression, thereby weakening the protective effects of estrogen on brain regions such as the hippocampus [36,37]. However, due to the small sample sizes in each subgroup after stratification, this finding requires further validation in studies with larger sample sizes.

Gestational glucose metabolism indicators may influence early childhood emotional and behavioral problems through several mechanisms. A key pathway is neuroinflammation, where persistent hyperglycemia activates immune responses and increases interleukin-6, disrupting central nervous system development [38,39]. In addition, intrauterine hyperglycemia may elevate placental vascular resistance, causing chronic fetal hypoxia and hippocampal injury, along with microangiopathy and endothelial dysfunction, leading to impaired nutrient and oxygen supply [40,41]. Hyperglycemia may also promote advanced glycation end product accumulation, inducing oxidative stress and neuroinflammation [42]. It may further downregulate CX3CR1, triggering microglial activation and abnormal synaptic pruning, thereby impairing neural plasticity [38]. At the epigenetic level, animal studies suggest that intrauterine hyperglycemia induces modifications in genes related to hippocampal synaptic plasticity and emotional regulation [43,44]. Neurotransmitter disturbances, particularly impaired serotonin signaling and dopaminergic dysfunction, may further contribute to cognitive and behavioral impairments [40,45,46].

PUFAs can mitigate the impact of abnormal glucose levels due to their antioxidant, anti-inflammatory, insulin-sensitizing properties and so on [47,48]. This study found that elevated levels of erythrocyte *n*-3 PUFAs can counteract the adverse association between maternal glucose metabolism and offspring emotional and behavioral problems, whereas *n*-6 PUFAs exhibit the opposite effect. Multiple previous studies have similarly reported that *n*-3 PUFAs can reduce the risk of diseases such as macrosomia and hyperbilirubinemia in the offspring of mothers with GDM [21,22]. Due to the competition between *n*-6 PUFAs and *n*-3 PUFAs during the desaturation process, focusing solely on the effects of either *n*-6 PUFAs or *n*-3 PUFAs on glucose metabolism has limited utility. Therefore, we also examined *n*-6/*n*-3 PUFA ratio but did not observe a significant impact on glucose metabolism-related emotional and behavioral problems in offspring. In our study, docosahexaenoic acid (DHA) accounted for the largest proportion of *n*-3 PUFAs. DHA and eicosapentaenoic acid (EPA) have been shown to inhibit NF-κB activation, thereby attenuating inflammation induced by hyperglycemia and insulin resistance and alleviating neuro-inflammatory damage in the hippocampus and prefrontal cortex [49,50,51]. In contrast, Arachidonic acid (AA) was the predominant component of *n*-6 PUFAs, which may promote the release of pro-inflammatory cytokines and exacerbate hyperglycemia-induced inflammatory responses and neurodevelopmental impairments [52,53]. Moreover, *n*-3 PUFAs enhance insulin signaling by maintaining insulin receptor and IRS-1 phosphorylation, improving cerebral insulin sensitivity and synaptic function. Conversely, *n*-6 PUFAs may impair central insulin signaling, facilitating brain insulin resistance and contributing to attentional deficits [54].

## 5. Strengths and Limitations

This study systematically included multiple maternal glucose metabolism indicators to provide a more comprehensive assessment of maternal glycemic status during pregnancy. To our knowledge, this is the first birth cohort study to investigate the effect of modification of erythrocyte PUFAs on the association between maternal glucose metabolism during pregnancy and offspring emotional and behavioral problems. Previous studies have found a positive correlation between dietary fatty acid intake and erythrocyte fatty acid levels [55,56]. The present findings suggest that increasing *n*-3 PUFAs intake (e.g., deep-sea fish) and limiting *n*-6 PUFAs-rich vegetable oils during early to mid-pregnancy may improve erythrocyte PUFA profiles and reduce the adverse impact of maternal hyperglycemia on offspring emotional and behavioral problems. These results highlight a potential nutritional strategy for offspring exposed to adverse intrauterine metabolic conditions.

However, several limitations should be acknowledged. First, observational studies cannot establish causal relationships between maternal glucose metabolic disorders and offspring emotional–behavioral problems. However, cohort study represents the highest hierarchy of evidence among observational designs. Second, while SDQ assessments may be subject to subjective bias, the SDQ scales are widely recognized for their strong reliability and validity and can be easily administered by trained professionals. Third, although residual confusions from unmeasured variables may introduce potential bias, baseline information, perinatal parameters, and longitudinal follow-up data in our cohort design minimize this risk. Additionally, some information collected during the survey, such as pre-pregnancy BMI, gestational weight gain, physical activity, and dietary energy intake, was based on self-reported recall, which may be subject to recall bias. This could introduce measurement error into these estimates. Fourth, we assessed erythrocytes PUFA levels only once during pregnancy. Although erythrocyte PUFA levels are generally considered stable and representative during the second trimester, fetal brain development occurs across all trimesters. Future studies with repeated PUFA measurements are needed to capture timing-specific effects at different gestational stages. Fifth, this study was conducted in a relatively developed urban area. Meanwhile, given the observed detrimental effects of HOMA-IR on offspring emotional and behavioral outcomes, and that this association was more evident among those with lower education levels, while participants who completed follow-up tended to have higher education levels, these factors may potentially skew the effect estimates toward zero. Therefore, caution should be exercised when generalizing our findings. Finally, the analyses of the five SDQ subscales were pre-specified as secondary, exploratory outcomes, and no multiplicity correction was applied. Future studies with larger samples are warranted to replicate and validate these associations.

## 6. Conclusions

Abnormal glucose metabolism during pregnancy, particularly FPG, OGTT-1h, and HOMA-IR, has been associated with an increased risk of emotional and behavioral problems in early childhood. The present findings indicate that elevated maternal erythrocytes *n*-3 PUFA levels and lower levels of *n*-6 PUFAs may mitigate the adverse associations between maternal glucose metabolism and early emotional–behavioral problems in children. Future studies with longer follow-up periods and exploring the potential effect-modifying roles of other fatty acid types are needed to confirm and extend these findings.

## Figures and Tables

**Table 1:** Maternal and children’s characteristics.

Characteristics	Total (*n* = 481)	GDM		*p* Value
		No (*n* = 388)	Yes (*n* = 93)	
Maternal age, y, mean (SD) †	30.41 ± 4.80	29.97 ± 4.66	32.24 ± 4.99	<0.001 *
Maternal educational level, *n* (%)				0.714
Below high school	68 (14.53%)	57 (15.16%)	11 (11.96%)	
High school to junior college	232 (49.57%)	186 (49.47%)	46 (50.00%)	
University and above	168 (35.90%)	133 (35.37%)	35 (38.04%)	
Pregnancy BMI, kg/m^2^, mean (SD) †	20.54 ± 2.86	20.31 ± 2.69	21.50 ± 3.30	0.003 *
Gestational weight gain, kg, mean (SD) †	14.16 ± 6.16	14.46 ± 6.10	12.91 ± 6.29	0.017 *
Family history of diabetes, *n* (%)				0.877
No	390 (83.16%)	313 (83.02%)	77 (83.70%)	
Yes	79 (16.84%)	64 (16.98%)	15 (16.30%)	
Maternal smoking, *n* (%)				0.091
No	340 (75.72%)	273 (74.59%)	67 (80.72%)	
Yes	98 (21.83%)	86 (23.50%)	12 (14.46%)	
Physical activity, h/d, mean (SD) †	1.25 ± 1.10	1.30 ± 1.15	1.07 ± 0.83	0.475
Dietary energy intake, kcal/d, mean (SD) †	1766.96 ± 485.17	1759.42 ± 491.82	1797.76 ± 458.34	0.337
Infant sex, *n* (%)				0.510
Male	216 (48.00%)	177 (48.76%)	39 (44.83%)	
Female	234 (52.00%)	186 (51.24%)	48 (55.17%)	
Mode of delivery, *n* (%)				0.009 *
Vaginal delivery	250 (63.13%)	209 (66.35%)	41 (50.62%)	
Cesarean section and others	146 (36.87%)	106 (33.65%)	40 (49.38%)	
Weeks of gestation, week, mean (SD) †	38.85 ± 1.29	38.92 ± 1.26	38.60 ± 1.37	0.148
Birth weight, kg, mean (SD) †	3.20 ± 0.40	3.20 ± 0.40	3.18 ± 0.41	0.514
Exclusive breastfeeding, *n* (%)				0.778
<4 months	169 (42.14%)	138 (42.59%)	31 (40.26%)	
4–6 months	87 (21.70%)	68 (20.99%)	19 (24.68%)	
≥6 months	145 (36.16%)	118 (36.42%)	27 (35.06%)	
complementary feeding age, *n* (%)				0.767
<6 months	212 (48.74%)	171 (48.58%)	41 (49.40%)	
≥6 months	216 (49.66%)	176 (50.00%)	40 (48.19%)	
Total difficulties, *n* (%)				0.640
Normal	417 (86.69%)	335 (86.34%)	82 (88.17%)	
Abnormal	64 (13.31%)	53 (13.66%)	11 (11.83%)	

* *p* < 0.05. † Variables with non-normal distribution. Data are presented as mean (SD) or *n* (%). *p* values were from *t* tests for normally distributed variables or Wilcoxon rank-sum tests for non-normally distributed variables, and χ2 tests for categorical variables. GDM, gestational diabetes mellitus; BMI, body mass index; SD, standard deviation.

**Table 2:** Glucose metabolic indicators and erythrocyte PUFAs level in pregnant women.

Variables	Total (*n* = 481)	GDM		*p* Value
		No (*n* = 388)	Yes (*n* = 93)	
Blood Glucose-Related Indicators				
FPG, mmol/L †	4.42 ± 0.42	4.33 ± 0.33	4.78 ± 0.54	<0.001 *
OGTT-1h glucose, mmol/L	7.82 ± 1.64	7.41 ± 1.36	9.51 ± 1.63	<0.001 *
OGTT-2h glucose, mmol/L †	6.74 ± 1.35	6.32 ± 0.99	8.53 ± 1.17	<0.001 *
HbA1c, %	5.06 ± 0.27	5.02 ± 0.26	5.25 ± 0.26	<0.001 *
Insulin-Related Indicators				
Insulin, IU †	4.26 ± 1.77	4.14 ±1.74	4.81 ± 1.78	0.002 *
HOMA-IR †	0.84 ± 0.38	0.80 ± 0.36	1.03 ± 0.41	<0.001 *
Erythrocyte PUFA Levels				
Total *n*-3 PUFAs, % †	9.70 ± 2.60	9.73 ± 2.60	9.55 ± 2.60	0.595
Total *n*-6 PUFAs, % †	35.04 ± 4.42	35.10 ± 4.41	34.79 ± 4.49	0.286
*n*-6: *n*-3 PUFAs †	3.91 ± 1.27	3.90 ± 1.28	3.94 ± 1.25	0.731

* *p* < 0.05. † Variables with non-normal distribution. Data are presented as mean (SD). *p* values were from *t* tests for normally distributed variables, or Wilcoxon rank-sum tests for non-normally distributed variables. PUFAs, polyunsaturated fatty acids; GDM, gestational diabetes mellitus; FPG, fasting plasma glucose; OGTT, oral glucose tolerance test; HbA1c, glycosylated hemoglobin; IU, International Units; HOMA-IR, HOMA-Insulin Resistance; SD, standard deviation.

**Table 3:** Association of glycemic metabolic indicators with emotional and behavioral problems in 5-year-old children (*n* = 481).

	OR (95%CI)					
	Emotional Symptoms	Conduct Problems	Hyperactivity /Inattention	Peer Relationship Problems	Prosocial Behavior	Total Difficulties Score
Model 1						
GDM	0.61 (0.22, 1.65)	1.16 (0.64, 2.08)	**2.21 (1.20, 4.10)**	0.90 (0.45, 1.77)	0.80 (0.33, 1.93)	1.34 (0.62, 2.91)
FPG, mmol/L	0.89 (0.61, 1.30)	1.18 (0.92, 1.53)	1.08 (0.82, 1.43)	1.18 (0.83, 1.67)	0.98 (0.74, 1.30)	1.32 (0.95, 1.85)
OGTT1h glucose, mmol/L	1.12 (0.78, 1.61)	1.19 (0.93, 1.53)	1.00 (0.77, 1.31)	0.79 (0.56, 1.11)	0.88 (0.67, 1.15)	**1.50 (1.07, 2.10)**
OGTT2h glucose, mmol/L	1.32 (0.93, 1.88)	1.17 (0.91, 1.50)	1.02 (0.78, 1.34)	0.90 (0.63, 1.28)	0.96 (0.73, 1.26)	1.24 (0.89, 1.72)
HbA1c, %	1.13 (0.77, 1.67)	1.24 (0.94, 1.64)	1.12 (0.83, 1.51)	1.25 (0.87, 1.79)	0.88 (0.66, 1.19)	1.12 (0.79, 1.58)
Insulin, IU	1.04 (0.73, 1.48)	1.07 (0.84, 1.38)	1.06 (0.81, 1.39)	0.85 (0.56, 1.28)	1.02 (0.76, 1.36)	1.36 (0.99, 1.87)
HOMA-IR	1.03 (0.71, 1.50)	1.10 (0.86, 1.42)	1.05 (0.80, 1.38)	0.86 (0.57, 1.31)	1.03 (0.76, 1.39)	**1.45 (1.05, 2.00)**
Model 2						
GDM	0.60 (0.22, 1.64)	1.02 (0.52, 2.00)	**2.05 (1.04, 4.04)**	0.98 (0.38, 2.51)	0.58 (0.25, 1.33)	1.73 (0.72, 4.17)
FPG, mmol/L	1.03 (0.70, 1.51)	1.10 (0.82, 1.47)	1.14 (0.84, 1.54)	1.23 (0.84, 1.81)	0.87 (0.63, 1.21)	**1.58 (1.07, 2.32) #**
OGTT1h glucose, mmol/L	1.04 (0.70, 1.53)	1.20 (0.90, 1.60)	1.09 (0.82, 1.46)	0.73 (0.49, 1.09)	0.75 (0.55, 1.02)	**1.58 (1.06, 2.35) #**
OGTT2h glucose, mmol/L	1.30 (0.90, 1.90)	1.24 (0.93, 1.65)	1.16 (0.86, 1.56)	0.88 (0.59, 1.31)	0.85 (0.62, 1.18)	1.37 (0.94, 2.01)
HbA1c, %	1.29 (0.85, 1.96)	**1.38 (1.01, 1.88)**	1.11 (0.81, 1.52)	1.51 (0.98, 2.31)	0.94 (0.68, 1.30)	1.29 (0.87, 1.92)
Insulin, IU	0.91 (0.56, 1.46)	1.12 (0.83, 1.52)	1.11 (0.81, 1.52)	0.59 (0.32, 1.07)	0.97 (0.67, 1.42)	1.49 (1.00, 2.23)
HOMA-IR	0.94 (0.59, 1.52)	1.14 (0.84, 1.54)	1.12 (0.82, 1.53)	0.64 (0.36, 1.14)	0.96 (0.65, 1.41)	**1.67 (1.12, 2.50) #**

Statistically significant (*p* < 0.05) results are in bold. # *p* < 0.1 after FDR correction. Model 1 of COX proportional hazard regression was adjusted for maternal age, maternal educational level, pregnancy BMI, gestational weight gain, family history of diabetes, maternal smoking, physical activity, dietary energy intake. Model 2 (main model) was adjusted for maternal age, maternal educational level, pregnancy BMI, gestational weight gain, family history of diabetes, maternal smoking, physical activity, dietary energy intake, children sex, mode of delivery, weeks of gestation, birth weight, exclusive breastfeeding and complementary feeding age. GDM, gestational diabetes mellitus; FPG, fasting plasma glucose; OGTT, oral glucose tolerance test; OR, odds ratio; CI, confidence interval; BMI, body mass index; HbA1c, glycosylated hemoglobin; HOMA-IR, HOMA-Insulin Resistance.

## Data Availability

Research data are not shared.

## Supplementary Materials

The following supporting information can be downloaded at https://www.mdpi.com/article/10.3390/nu18121840/s1, Table S1: Baseline characteristics of non-included and included populations; Figure S1: Nonlinear association between maternal blood glucose during pregnancy and offspring emotional and behavioral problems; Table S2: Interaction between maternal erythrocyte membrane PUFAs and glucose metabolism levels on offspring’s neuropsychological development; Table S3: Sensitivity analysis of the association between glycemic metabolic indicators and emotional and behavioral problems in full-term children at age 5; Table S4: Sensitivity analysis of the association between glycemic metabolic indicators and emotional and behavioral problems in normal-weight children at age 5; Table S5: Sensitivity analysis of the association between glycemic metabolic indicators and emotional and behavioral problems in 5-year-old children, adjusted for disease in offspring at age 6; Table S6: Sensitivity analysis of the association between glycemic metabolic indicators and emotional and behavioral problems in 5-year-old children, adjusted for outdoor activities in offspring at age 6; Table S7: Stratified associations between maternal glucose metabolic markers and offspring total difficulties scores by child sex.

## References

[1] Egger H.L., Angold A. (2006). Common emotional and behavioral disorders in preschool children: Presentation, nosology, and epidemiology. J. Child Psychol. Psychiatry.

[2] Polanczyk G.V., Salum G.A., Sugaya L.S., Caye A., Rohde L.A. (2015). Annual research review: A meta-analysis of the worldwide prevalence of mental disorders in children and adolescents. J. Child Psychol. Psychiatry.

[3] Liu W.W., Wu X.Y., Tao S.M., Ding P., Geng M.L., Tao F.B. (2020). Emotional and behavioral problems associated with health-risk behaviors in preschool children. Zhonghua Yu Fang Yi Xue Za Zhi.

[4] Fernández de Cossío L., Lacabanne C., Bordeleau M., Castino G., Kyriakakis P., Tremblay M. (2021). Lipopolysaccharide-induced maternal immune activation modulates microglial CX3CR1 protein expression and morphological phenotype in the hippocampus and dentate gyrus, resulting in cognitive inflexibility during late adolescence. Brain Behav. Immun..

[5] Wang P., Xie J., Jiao X.C., Ma S.S., Liu Y., Yin W.J., Tao R.X., Hu H.L., Zhang Y., Chen X.X. (2021). Maternal Glycemia During Pregnancy and Early Offspring Development: A Prospective Birth Cohort Study. J. Clin. Endocrinol. Metab..

[6] Wang H., Li N., Chivese T., Werfalli M., Sun H., Yuen L., Hoegfeldt C.A., Elise Powe C., Immanuel J., Karuranga S. (2022). IDF Diabetes Atlas: Estimation of Global and Regional Gestational Diabetes Mellitus Prevalence for 2021 by International Association of Diabetes in Pregnancy Study Group’s Criteria. Diabetes Res. Clin. Pract..

[7] DF Diabetes Atlas (2025). Global Diabetes Data & Insights.

[8] Faleschini S., Doyon M., Arguin M., Lepage J.F., Tiemeier H., Van Lieshout R.J., Perron P., Bouchard L., Hivert M.F. (2023). Maternal Hyperglycemia in Pregnancy and Offspring Internalizing and Externalizing Behaviors. Matern. Child. Health J..

[9] Pretorius R.A., Avraam D., Guxens M., Julvez J., Harris J.R., Nader J.T., Cadman T., Elhakeem A., Strandberg-Larsen K., Marroun H.E. (2025). Is maternal diabetes during pregnancy associated with neurodevelopmental, cognitive and behavioural outcomes in children? Insights from individual participant data meta-analysis in ten birth cohorts. BMC Pediatr..

[10] Mattila I., Nolvi S., Kataja E.L., Tuulari J.J., Korja R., Scheinin N.M., Kaaja R., Karlsson H., Ekholm E., Karlsson L. (2026). Gestational diabetes mellitus and children’s social-emotional development, behavioral problems, and psychological adjustment. Pediatr. Res..

[11] Cendra-Duarte E., Canals J., Becerra-Tomás N., Mateu-Fabregat J., Bulló M., Arija V. (2025). Dietary glycemic index and load during pregnancy and offspring behavioral outcomes: Exploring sex differences. Eur. J. Pediatr..

[12] Wan C.S., Abell S., Aroni R., Nankervis A., Boyle J., Teede H. (2019). Ethnic differences in prevalence, risk factors, and perinatal outcomes of gestational diabetes mellitus: A comparison between immigrant ethnic Chinese women and Australian-born Caucasian women in Australia. J. Diabetes.

[13] Metzger B.E., Lowe L.P., Dyer A.R., Trimble E.R., Chaovarindr U., Coustan D.R., Hadden D.R., McCance D.R., Hod M., McIntyre H.D. (2008). Hyperglycemia and adverse pregnancy outcomes. N. Engl. J. Med..

[14] Papachatzopoulou E., Chatzakis C., Lambrinoudaki I., Panoulis K., Dinas K., Vlahos N., Sotiriadis A., Eleftheriades M. (2020). Abnormal fasting, post-load or combined glucose values on oral glucose tolerance test and pregnancy outcomes in women with gestational diabetes mellitus. Diabetes Res. Clin. Pract..

[15] Wang C.S., Wei Y.M., Yang H.X. (2013). Analysis of the effects of gestational diabetes mellitus based on abnormal blood glucose on pregnancy outcomes. Zhonghua Fu Chan Ke Za Zhi.

[16] Chatzakis C., Eleftheriades A., Demertzidou E., Dinas K., Vlahos N., Sotiriadis A., Eleftheriades M. (2023). Pregnancy outcomes in the different phenotypes of gestational diabetes mellitus based on the oral glucose tolerance test. A systematic review and meta-analysis. Diabetes Res. Clin. Pract..

[17] Oliver E., McGillicuddy F.C., Harford K.A., Reynolds C.M., Phillips C.M., Ferguson J.F., Roche H.M. (2012). Docosahexaenoic acid attenuates macrophage-induced inflammation and improves insulin sensitivity in adipocytes-specific differential effects between LC *n*-3 PUFA. J. Nutr. Biochem..

[18] Innis S.M. (2008). Dietary omega 3 fatty acids and the developing brain. Brain Res..

[19] Gao L., Lin L., Shan N., Ren C.Y., Long X., Sun Y.H., Wang L. (2020). The impact of omega-3 fatty acid supplementation on glycemic control in patients with gestational diabetes: A systematic review and meta-analysis of randomized controlled studies. J. Matern. Fetal Neonatal Med..

[20] Carbone B.E., Abouleish M., Watters K.E., Vogel S., Ribic A., Schroeder O.H., Bader B.M., Biederer T. (2020). Synaptic Connectivity and Cortical Maturation Are Promoted by the ω-3 Fatty Acid Docosahexaenoic Acid. Cereb. Cortex.

[21] Jamilian M., Hashemi Dizaji S., Bahmani F., Taghizadeh M., Memarzadeh M.R., Karamali M., Akbari M., Asemi Z. (2017). A Randomized Controlled Clinical Trial Investigating the Effects of Omega-3 Fatty Acids and Vitamin E Co-Supplementation on Biomarkers of Oxidative Stress, Inflammation and Pregnancy Outcomes in Gestational Diabetes. Can. J. Diabetes.

[22] Khan N.A. (2007). Role of lipids and fatty acids in macrosomic offspring of diabetic pregnancy. Cell Biochem. Biophys..

[23] Metzger B.E., Gabbe S.G., Persson B., Buchanan T.A., Catalano P.A., Damm P., Dyer A.R., de Leiva A., Hod M., Kitzmiler J.L. (2010). International association of diabetes and pregnancy study groups recommendations on the diagnosis and classification of hyperglycemia in pregnancy. Diabetes Care.

[24] Matthews D.R., Hosker J.P., Rudenski A.S., Naylor B.A., Treacher D.F., Turner R.C. (1985). Homeostasis model assessment: Insulin resistance and beta-cell function from fasting plasma glucose and insulin concentrations in man. Diabetologia.

[25] Du Y., Kou J., Coghill D. (2008). The validity, reliability and normative scores of the parent, teacher and self report versions of the Strengths and Difficulties Questionnaire in China. Child. Adolesc. Psychiatry Ment. Health.

[26] Goodman A., Goodman R. (2011). Population mean scores predict child mental disorder rates: Validating SDQ prevalence estimators in Britain. J. Child Psychol. Psychiatry.

[27] Tao H., Shao T., Ni L., Sun Y., Yan S., Gu C., Cao H., Huang K., Tao F., Tong S. (2016). The relationship between maternal emotional symptoms during pregnancy and emotional and behavioral problems in preschool children: A birth cohort study. Zhonghua Yu Fang Yi Xue Za Zhi.

[28] Peng S., Du Z., He Y., Zhao F., Chen Y., Wu S., Hao Y., Cai L. (2022). Association of Maternal Erythrocyte PUFA during Pregnancy with Offspring Allergy in the Chinese Population. Nutrients.

[29] Shuffrey L.C., Morales S., Jacobson M.H., Bosquet Enlow M., Ghassabian A., Margolis A.E., Lucchini M., Carroll K.N., Crum R.M., Dabelea D. (2023). Association of Gestational Diabetes Mellitus and Perinatal Maternal Depression with Early Childhood Behavioral Problems: An Environmental Influences on Child Health Outcomes (ECHO) Study. Child. Dev..

[30] Titmuss A., D’Aprano A., Barzi F., Brown A.D.H., Wood A., Connors C., Boyle J.A., Moore E., O’Dea K., Oats J. (2022). Hyperglycemia in pregnancy and developmental outcomes in children at 18–60 months of age: The PANDORA Wave 1 study. J. Dev. Orig. Health Dis..

[31] Daraki V., Roumeliotaki T., Koutra K., Georgiou V., Kampouri M., Kyriklaki A., Vafeiadi M., Papavasiliou S., Kogevinas M., Chatzi L. (2017). Effect of parental obesity and gestational diabetes on child neuropsychological and behavioral development at 4 years of age: The Rhea mother-child cohort, Crete, Greece. Eur. Child. Adolesc. Psychiatry.

[32] Zhen H., Chen S., Zhang M., Liu C., Tong J., Gao G., Wu X., Gan H., Yan S., Tao F. (2025). Sex-specific association between maternal integrative endocrine and metabolic status during pregnancy and preschoolers’ emotional-behavioral development: A birth cohort study. Eur. Child. Adolesc. Psychiatry.

[33] Wallace T.M., Levy J.C., Matthews D.R. (2004). Use and abuse of HOMA modeling. Diabetes Care.

[34] Sherwani S.I., Khan H.A., Ekhzaimy A., Masood A., Sakharkar M.K. (2016). Significance of HbA1c Test in Diagnosis and Prognosis of Diabetic Patients. Biomark. Insights.

[35] Sun H.L., He F., Rao W.W., Qi Y., Rao S.Y., Ho T.I., Su Z., Cheung T., Wong K.K., Smith R.D. (2025). Gender differences in behavioral and emotional problems among school children and adolescents in China: National survey findings from a comparative network perspective. J. Affect. Disord..

[36] Scharfman H.E., MacLusky N.J. (2006). Estrogen and brain-derived neurotrophic factor (BDNF) in hippocampus: Complexity of steroid hormone-growth factor interactions in the adult CNS. Front. Neuroendocrinol..

[37] Stern C., Schwarz S., Moser G., Cvitic S., Jantscher-Krenn E., Gauster M., Hiden U. (2021). Placental Endocrine Activity: Adaptation and Disruption of Maternal Glucose Metabolism in Pregnancy and the Influence of Fetal Sex. Int. J. Mol. Sci..

[38] Vuong B., Odero G., Rozbacher S., Stevenson M., Kereliuk S.M., Pereira T.J., Dolinsky V.W., Kauppinen T.M. (2017). Exposure to gestational diabetes mellitus induces neuroinflammation, derangement of hippocampal neurons, and cognitive changes in rat offspring. J. Neuroinflammation.

[39] Piazza F.V., Segabinazi E., de Meireles A.L.F., Mega F., Spindler C.F., Augustin O.A., Salvalaggio G.D.S., Achaval M., Kruse M.S., Coirini H. (2019). Severe Uncontrolled Maternal Hyperglycemia Induces Microsomia and Neurodevelopment Delay Accompanied by Apoptosis, Cellular Survival, and Neuroinflammatory Deregulation in Rat Offspring Hippocampus. Cell Mol. Neurobiol..

[40] Rodolaki K., Pergialiotis V., Iakovidou N., Boutsikou T., Iliodromiti Z., Kanaka-Gantenbein C. (2023). The impact of maternal diabetes on the future health and neurodevelopment of the offspring: A review of the evidence. Front. Endocrinol..

[41] Xiang A.H., Wang X., Martinez M.P., Getahun D., Page K.A., Buchanan T.A., Feldman K. (2018). Maternal Gestational Diabetes Mellitus, Type 1 Diabetes, and Type 2 Diabetes During Pregnancy and Risk of ADHD in Offspring. Diabetes Care.

[42] Chandna A.R., Kuhlmann N., Bryce C.A., Greba Q., Campanucci V.A., Howland J.G. (2015). Chronic maternal hyperglycemia induced during mid-pregnancy in rats increases RAGE expression, augments hippocampal excitability, and alters behavior of the offspring. Neuroscience.

[43] Zou K., Ren J., Luo S., Zhang J., Zhou C., Tan C., Lv P., Sun X., Sheng J., Liu X. (2021). Intrauterine hyperglycemia impairs memory across two generations. Transl. Psychiatry.

[44] Fusco S., Spinelli M., Cocco S., Ripoli C., Mastrodonato A., Natale F., Rinaudo M., Livrizzi G., Grassi C. (2019). Maternal insulin resistance multigenerationally impairs synaptic plasticity and memory via gametic mechanisms. Nat. Commun..

[45] Martin H., Bullich S., Martinat M., Chataigner M., Di Miceli M., Simon V., Clark S., Butler J., Schell M., Chopra S. (2024). Insulin modulates emotional behavior through a serotonin-dependent mechanism. Mol. Psychiatry.

[46] Kleinridders A., Cai W., Cappellucci L., Ghazarian A., Collins W.R., Vienberg S.G., Pothos E.N., Kahn C.R. (2015). Insulin resistance in brain alters dopamine turnover and causes behavioral disorders. Proc. Natl. Acad. Sci. USA.

[47] Chen Y.J., Lin L.Z., Liu Z.Y., Wang X., Karatela S., Wang Y.X., Peng S.S., Jiang B.B., Li X.X., Liu N. (2023). Association between maternal gestational diabetes and allergic diseases in offspring: A birth cohort study. World J. Pediatr..

[48] Steenweg-de Graaff J.C., Tiemeier H., Basten M.G., Rijlaarsdam J., Demmelmair H., Koletzko B., Hofman A., Jaddoe V.W., Verhulst F.C., Roza S.J. (2015). Maternal LC-PUFA status during pregnancy and child problem behavior: The Generation R Study. Pediatr. Res..

[49] Calder P.C. (2013). Omega-3 polyunsaturated fatty acids and inflammatory processes: Nutrition or pharmacology?. Br. J. Clin. Pharmacol..

[50] Calder P.C. (2020). *n*-3 PUFA and inflammation: From membrane to nucleus and from bench to bedside. Proc. Nutr. Soc..

[51] Calder P.C. (2010). Omega-3 fatty acids and inflammatory processes. Nutrients.

[52] Zhang Y., Liu Y., Sun J., Zhang W., Guo Z., Ma Q. (2023). Arachidonic acid metabolism in health and disease. MedComm.

[53] Dennis E.A., Norris P.C. (2015). Eicosanoid storm in infection and inflammation. Nat. Rev. Immunol..

[54] Taouis M., Dagou C., Ster C., Durand G., Pinault M., Delarue J. (2002). *N*-3 polyunsaturated fatty acids prevent the defect of insulin receptor signaling in muscle. Am. J. Physiol. Endocrinol. Metab..

[55] Sun Q., Ma J., Campos H., Hankinson S.E., Hu F.B. (2007). Comparison between plasma and erythrocyte fatty acid content as biomarkers of fatty acid intake in US women. Am. J. Clin. Nutr..

[56] Huang Z., Guo P., Wang Y., Li Z., Yin X., Chen M., Liu Y., Hu Y., Chen B. (2022). Docosahexaenoic Acid as the Bidirectional Biomarker of Dietary and Metabolic Risk Patterns in Chinese Children: A Comparison with Plasma and Erythrocyte. Nutrients.

