# Supplementary material for: Maternal Glucose Metabolism and Emotional and Behavioral Problems in Offspring: Modification by Erythrocyte Polyunsaturated Fatty Acids
Source: Nutrients. 2026 Jun 6;18(12):1840. doi: 10.3390/nu18121840 (PMC13305560; doi:10.3390/nu18121840)
Supplement: Supplementary file 1 [file nutrients-18-01840-s001.zip › nutrients-4296978-supplementary.pdf]

Table S1 Baseline characteristics of non-included and included populations

Characteristics	Non-included （*n*=515）	Included （*n*=481）	*P* value
**Maternal age, y, mean (SD[a])**	30.10 ± 5.02	30.39 ± 4.84	0.342
**Maternal educational level, n (%)**			**0.006***
below high school	95 (19.35%)	68 (14.59%)	
high school to junior college	264 (53.77%)	232 (49.57%)	
University and above	132 (26.88%)	168 (35.84%)	
**Pregnancy BMI, kg/m², mean (SD)**	20.65 ± 3.14	20.54 ± 2.86	0.577
**Child's sex, n (%)**			0.229
Male	226 (51.48%)	216 (48.00%)	
Female	213 (48.52%)	234 (52.00%)	

*$P<0.05$, Statistically significant ($P<0.05$) results were in bold. P values were from t tests or $\chi 2$ tests.

[a] Data were presented as mean (SD) or n (%). SD, standard deviation.

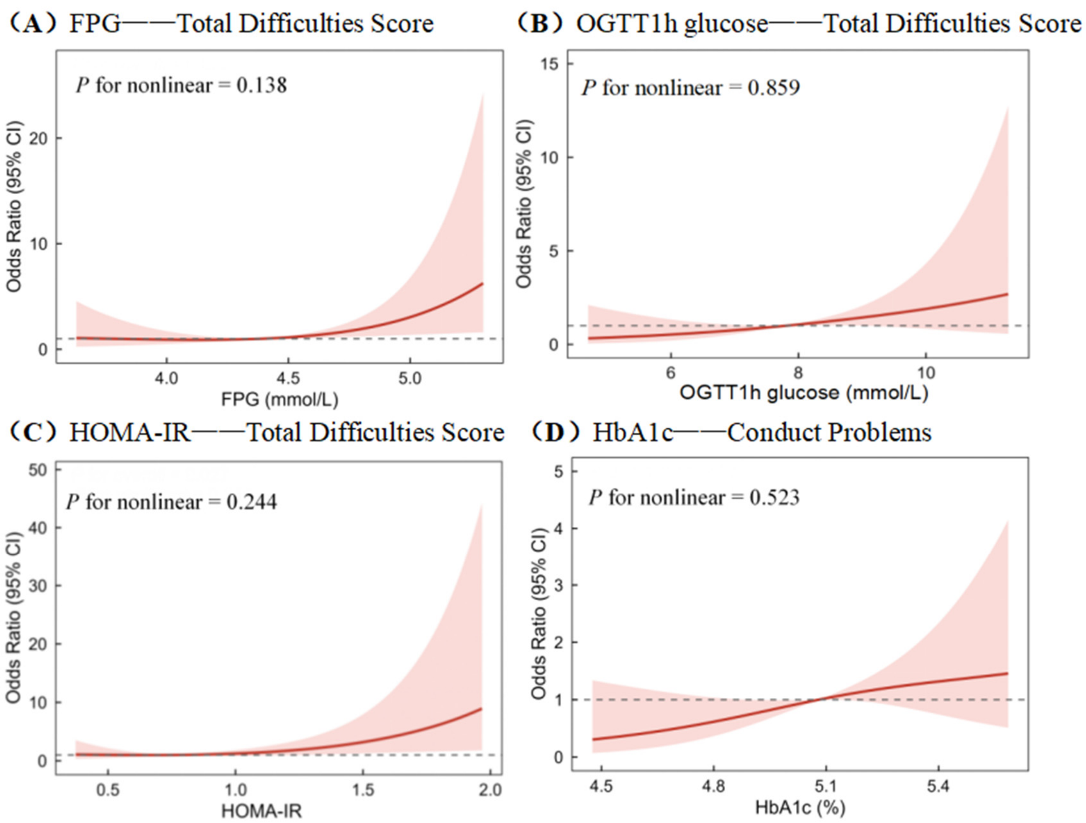

Figure S1 Nonlinear association between maternal blood glucose during pregnancy and offspring emotional and behavioral problems

Table S2 Interaction between maternal erythrocyte membrane PUFAs and glucose metabolism levels on offspring's neuropsychological development.（*n*=481）

	***OR*（95%*CI*）**					
	**Emotional Symptoms**	**Conduct Problems**	**Hyperactivity/Inattention**	**Peer Relationship Problems**	**Prosocial Behavior**	**Total Difficulties Score**
**n-3 PUFAs**						
FPG, mmol/L	1.01 (0.64, 1.61)	0.84 (0.59, 1.20)	0.91 (0.65, 1.33)	0.75 (0.49, 1.15)	0.66 (0.38, 1.15)	**0.52 (0.30, 0.91)***
OGTT1h glucose, mmol/L	1.07 (0.70, 1.62)	0.96 (0.71, 1.29)	0.83 (0.62, 1.11)	1.13 (0.81, 1.59)	0.95 (0.59, 1.53)	0.98 (0.63, 1.53)
OGTT2h glucose, mmol/L	0.92 (0.60, 1.43)	0.91 (0.67, 1.23)	0.91 (0.67, 1.24)	1.01 (0.70, 1.46)	1.53 (0.92, 2.55)	0.79 (0.50, 1.24)
HbA1c, %	0.92 (0.54, 1.56)	0.87 (0.60, 1.27)	1.06 (0.72, 1.57)	1.39 (0.91, 2.12)	0.84 (0.48, 1.48)	0.63 (0.37, 1.07)
Insulin, IU	0.80 (0.48, 1.33)	0.73 (0.52, 1.03)	1.03 (0.73, 1.45)	0.71 (0.48, 1.07)	1.33 (0.56, 3.14)	0.69 (0.34, 1.23)
HOMA-IR	0.80 (0.48, 1.34)	0.70 (0.49, 1.00)	0.99 (0.70, 1.40)	0.67 (0.43, 1.03)	1.04 (0.46, 2.39)	**0.51 (0.30, 0.91)***
**n-6 PUFAs**						
FPG, mmol/L	1.01 (0.60, 1.68)	0.86 (0.59, 1.27)	**1.63 (1.05, 2.51)***	0.92 (0.62, 1.37)	1.41 (0.82, 2.44)	0.95 (0.56, 1.62)
OGTT1h glucose, mmol/L	1.06 (0.64, 1.76)	0.85 (0.59, 1.24)	0.91 (0.67, 1.28)	0.80 (0.56, 1.13)	1.43 (0.77, 2.64)	0.73 (0.42, 1.28)
OGTT2h glucose, mmol/L	1.01 (0.56, 1.84)	0.87 (0.57, 1.32)	1.30 (0.86, 1.98)	0.88 (0.55, 1.39)	1.27 (0.65, 2.47)	0.75 (0.40, 1.41)
HbA1c, %	0.73 (0.40, 1.36)	1.20 (0.78, 1.84)	1.18 (0.79, 1.74)	1.21 (0.80, 1.85)	0.99 (0.55, 1.80)	1.45 (0.75, 2.81)
Insulin, IU	0.79 (0.46, 1.36)	0.78 (0.53, 1.14)	0.84 (0.58, 1.20)	0.99 (0.62, 1.57)	2.18 (0.74, 6.45)	0.69 (0.39, 1.23)
HOMA-IR	0.81 (0.48, 1.36)	0.79 (0.55, 1.13)	0.93 (0.66, 1.32)	0.97 (0.62, 1.53)	2.45 (0.80, 7.45)	0.74 (0.44, 1.24)
**n-6/n-3 PUFAs**						
FPG, mmol/L	0.96 (0.59, 1.55)	1.01 (0.72, 1.42)	1.20 (0.84, 1.73)	1.16 (0.78, 1.71)	1.64 (0.95, 2.85)	1.72 (0.95, 3.13)
OGTT1h glucose, mmol/L	0.87 (0.56, 1.35)	1.01 (0.75, 1.35)	1.19 (0.88, 1.60)	0.80 (0.56, 1.13)	1.23 (0.81, 1.87)	0.95 (0.60, 1.52)
OGTT2h glucose, mmol/L	1.09 (0.68, 1.74)	1.10 (0.80, 1.52)	1.17 (0.84, 1.63)	0.93 (0.63, 1.38)	0.66 (0.38, 1.15)	1.19 (0.73, 1.95)
HbA1c, %	1.00 (0.61, 1.63)	1.09 (0.75, 1.57)	0.94 (0.67, 1.32)	0.79 (0.54, 1.16)	1.01 (0.62, 1.64)	1.72 (0.93, 3.19)
Insulin, IU	1.12 (0.73, 1.70)	1.17 (0.89, 1.54)	0.86 (0.62, 1.20)	1.24 (0.91, 1.68)	0.87 (0.39, 1.91)	1.18 (0.80, 1.74)

HOMA-IR	1.12 (0.71, 1.75)	1.19 (0.89, 1.59)	0.91 (0.66, 1.26)	1.27 (0.92, 1.80)	1.05 (0.53, 2.09)	1.29 (0.84, 1.98)

*$P<0.05$, Statistically significant ($P<0.05$) results were in bold.

[a] The main model was adjusted for maternal age, maternal educational level, pregnancy BMI, gestational weight gain, family history of diabetes, maternal smoking, physical activity, dietary energy intake, child's sex, mode of delivery, weeks of gestation, birthweight, exclusive breastfeeding and complementary feeding age. GDM, gestational diabetes mellitus; FPG, Fasting plasma glucose; OGTT, oral glucose tolerance test; OR, Odds Ratio; CI, confidence interval; BMI, body mass index; HbA1C, glycosylated hemoglobin; HOMA-IR, HOMA-Insulin Resistance.

Table S3 Sensitivity analysis of the association between glycemic metabolic indicators and emotional and behavioral problems in full-term children at age 5.（*n*=378）

	OR（95%*CI*）					
	**Emotional Symptoms**	**Conduct Problems**	**Hyperactivity/Inattention**	**Peer Relationship Problems**	**Prosocial Behavior**	**Total Difficulties Score**
**GDM[a]**	0.65 (0.23, 1.81)	1.13 (0.57, 2.26)	**2.07 (1.03, 4.16)***	0.87 (0.32, 2.35)	0.55 (0.23, 1.33)	1.86 (0.77, 4.51)
**FPG, mmol/L**	1.05 (0.71, 1.55)	1.13 (0.84, 1.52)	1.15 (0.84, 1.56)	1.29 (0.88, 1.89)	0.87 (0.62, 1.22)	**1.60 (1.08, 2.37)***
**OGTT1h glucose, mmol/L**	1.03 (0.70, 1.53)	1.23 (0.91, 1.65)	1.08 (0.80, 1.45)	0.70 (0.46, 1.05)	0.75 (0.54, 1.02)	**1.58 (1.05, 2.37)***
**OGTT2h glucose, mmol/L**	1.31 (0.89, 1.91)	1.24 (0.93, 1.66)	1.10 (0.82, 1.49)	0.81 (0.54, 1.23)	0.86 (0.61, 1.20)	1.37 (0.93, 2.01)
**HbA1c, percent**	1.28 (0.84, 1.95)	1.36 (0.99, 1.88)	1.03 (0.74, 1.42)	1.42 (0.92, 2.18)	0.96 (0.68, 1.35)	1.24 (0.83, 1.85)
**Insulin, IU**	0.89 (0.55, 1.43)	1.11 (0.82, 1.50)	1.11 (0.81, 1.53)	0.55 (0.30, 1.03)	1.04 (0.71, 1.52)	1.53 (1.02, 2.29)
**HOMA-IR**	0.93 (0.58, 1.49)	1.12 (0.83, 1.52)	1.12 (0.82, 1.53)	0.63 (0.35, 1.13)	1.02 (0.69, 1.51)	**1.70 (1.14, 2.55)***

**P*<0.05, Statistically significant (*P*<0.05) results were in bold.

[a] The main model was adjusted for maternal age, maternal educational level, pregnancy BMI, gestational weight gain, family history of diabetes, maternal smoking, physical activity, dietary energy intake, child's sex, mode of delivery, birthweight, exclusive breastfeeding and complementary feeding age. GDM, gestational diabetes mellitus; FPG, Fasting plasma glucose; OGTT, oral glucose tolerance test; OR, Odds Ratio; CI, confidence interval; BMI, body mass index; HbA1C, glycosylated hemoglobin; HOMA-IR, HOMA-Insulin Resistance.

Table S4 Sensitivity analysis of the association between glycemic metabolic indicators and emotional and behavioral problems in normal-weight children at age 5.（*n*=373）

	***OR*（95%*CI*）**					
	**Emotional Symptoms**	**Conduct Problems**	**Hyperactivity/Inattention**	**Peer Relationship Problems**	**Prosocial Behavior**	**Total Difficulties Score**
**GDM[a]**	0.61 (0.22, 1.70)	1.08 (0.54, 2.17)	**2.23 (1.09, 4.53)***	0.80 (0.28, 2.27)	0.60 (0.26, 1.40)	1.79 (0.73, 4.36)
**FPG, mmol/L**	1.05 (0.72, 1.53)	1.12 (0.84, 1.51)	1.19 (0.87, 1.62)	1.16 (0.78, 1.72)	0.88 (0.63, 1.20)	**1.57 (1.07, 2.32)***
**OGTT1h glucose, mmol/L**	1.10 (0.74, 1.62)	1.22 (0.91, 1.64)	1.13 (0.84, 1.52)	0.68 (0.45, 1.04)	0.77 (0.56, 1.05)	**1.62 (1.08, 2.41)***
**OGTT2h glucose, mmol/L**	1.41 (0.96, 2.06)	1.27 (0.95, 1.71)	1.17 (0.86, 1.59)	0.72 (0.46, 1.13)	0.89 (0.64, 1.24)	1.46 (0.99, 2.15)
**HbA1c, %**	1.24 (0.80, 1.92)	1.33 (0.96, 1.83)	1.06 (0.76, 1.47)	1.31 (0.84, 2.04)	0.90 (0.64, 1.25)	1.25 (0.83, 1.89)
**Insulin, IU**	0.86 (0.53, 1.41)	1.16 (0.84, 1.59)	1.17 (0.84, 1.63)	0.56 (0.30, 1.04)	0.94 (0.65, 1.38)	1.50 (0.99, 2.29)
**HOMA-IR**	0.91 (0.56, 1.47)	1.18 (0.85, 1.64)	1.20 (0.85, 1.69)	0.58 (0.31, 1.08)	0.93 (0.63, 1.37)	**1.73 (1.13, 2.65)***

*P<0.05, Statistically significant (P<0.05) results were in bold.

[a] The main model was adjusted for maternal age, maternal educational level, pregnancy BMI, gestational weight gain, family history of diabetes, maternal smoking, physical activity, dietary energy intake, child's sex, mode of delivery, weeks of gestation, exclusive breastfeeding and complementary feeding age. GDM, gestational diabetes mellitus; FPG, Fasting plasma glucose; OGTT, oral glucose tolerance test; OR, Odds Ratio; CI, confidence interval; BMI, body mass index; HbA1C, glycosylated hemoglobin; HOMA-IR, HOMA-Insulin Resistance.

Table S5 Sensitivity analysis of the association between glycemic metabolic indicators and emotional and behavioral problems in 5-Year-Old Children, Adjusted for disease in offspring at age 6.（*n*=481）

	***OR*（95%*CI*）**					
	**Emotional Symptoms**	**Conduct Problems**	**Hyperactivity/Inattention**	**Peer Relationship Problems**	**Prosocial Behavior**	**Total Difficulties Score**
**GDM[a]**	0.59 (0.22, 1.60)	1.00 (0.51, 1.97)	**2.05 (1.04, 4.04)***	0.96 (0.37, 2.45)	0.57 (0.25, 1.31)	1.70 (0.71, 4.03)
**FPG,mmol/L**	1.01 (0.69, 1.49)	1.13 (0.84, 1.52)	1.15 (0.85, 1.56)	1.24 (0.85, 1.82)	0.85 (0.61, 1.18)	**1.55 (1.05, 2.28)***
**OGTT1h glucose, mmol/L**	1.06 (0.72, 1.57)	1.19 (0.90, 1.59)	1.08 (0.81, 1.45)	0.72 (0.48, 1.07)	0.76 (0.56, 1.04)	**1.64 (1.10, 2.46)***
**OGTT2h glucose, mmol/L**	1.29 (0.89, 1.86)	1.26 (0.94, 1.68)	1.16 (0.86, 1.56)	0.88 (0.59, 1.30)	0.85 (0.62, 1.18)	1.35 (0.92, 1.96)
**HbA1c, %**	1.29 (0.85, 1.96)	**1.41 (1.03, 1.93)***	1.11 (0.81, 1.52)	1.49 (0.97, 2.27)	0.94 (0.68, 1.31)	1.29 (0.87, 1.93)
**Insulin, IU**	0.91 (0.56, 1.46)	1.15 (0.85, 1.55)	1.11 (0.81, 1.51)	0.60 (0.33, 1.08)	0.98 (0.68, 1.43)	1.50 (1.00, 2.25)
**HOMA-IR**	0.94 (0.59, 1.52)	1.16 (0.86, 1.57)	1.12 (0.83, 1.53)	0.66 (0.37, 1.15)	0.96 (0.66, 1.42)	**1.66 (1.11, 2.49)***

*P<0.05, Statistically significant (P<0.05) results were in bold.

[a] The main model was adjusted for maternal age, maternal educational level, pregnancy BMI, gestational weight gain, family history of diabetes, maternal smoking, physical activity, dietary energy intake, child's sex, mode of delivery, weeks of gestation, birthweight, exclusive breastfeeding complementary feeding age and disease in offspring at age 6. GDM, gestational diabetes mellitus; FPG, Fasting plasma glucose; OGTT, oral glucose tolerance test; OR, Odds Ratio; CI, confidence interval; BMI, body mass index; HbA1C, glycosylated hemoglobin; HOMA-IR, HOMA-Insulin Resistance.

Table S6 Sensitivity analysis of the association between glycemic metabolic indicators and emotional and behavioral problems in 5-Year-Old Children, Adjusted for outdoor activities in offspring at age 6.（*n*=481）

	***OR*（95%*CI*）**					
	**Emotional Symptoms**	**Conduct Problems**	**Hyperactivity/Inattention**	**Peer Relationship Problems**	**Prosocial Behavior**	**Total Difficulties Score**
**GDM[a]**	0.87 (0.31, 2.44)	0.94 (0.46, 1.96)	**2.12 (1.02, 4.42)***	0.93 (0.34, 2.51)	0.62 (0.25, 1.50)	2.00 (0.76, 5.27)
**FPG,mmol/L**	1.11 (0.74, 1.67)	1.16 (0.84, 1.60)	1.22 (0.88, 1.70)	1.38 (0.91, 2.10)	0.79 (0.55, 1.14)	**2.20 (1.35, 3.56)***
**OGTT1h glucose, mmol/L**	1.05 (0.68, 1.62)	1.17 (0.86, 1.61)	1.06 (0.78, 1.46)	0.68 (0.44, 1.03)	0.79 (0.56, 1.11)	**1.95 (1.21, 3.14)***
**OGTT2h glucose, mmol/L**	1.45 (0.97, 2.19)	1.18 (0.87, 1.61)	1.16 (0.84, 1.59)	0.81 (0.54, 1.24)	0.93 (0.66, 1.33)	**1.74 (1.15, 2.63)***
**HbA1c, %**	1.32 (0.82, 2.12)	**1.49 (1.04, 2.12)***	1.06 (0.75, 1.50)	1.31 (0.83, 2.05)	1.05 (0.72, 1.52)	1.52 (0.97, 2.37)
**Insulin, IU**	0.86 (0.50, 1.48)	0.97 (0.70, 1.35)	0.91 (0.64, 1.31)	0.54 (0.28, 1.04)	0.84 (0.54, 1.30)	1.46 (0.94, 2.27)
**HOMA-IR**	0.95 (0.56, 1.61)	0.98 (0.71, 1.35)	0.96 (0.68, 1.36)	0.60 (0.32, 1.15)	0.80 (0.51, 1.26)	**1.69 (1.09, 2.62)***

*P<0.05, Statistically significant (P<0.05) results were in bold.

[a] The main model was adjusted for maternal age, maternal educational level, pregnancy BMI, gestational weight gain, family history of diabetes, maternal smoking, physical activity, dietary energy intake, child's sex, mode of delivery, weeks of gestation, birthweight, exclusive breastfeeding complementary feeding age and outdoor activities.

GDM, gestational diabetes mellitus; FPG, Fasting plasma glucose; OGTT, oral glucose tolerance test; OR, Odds Ratio; CI, confidence interval; BMI, body mass index; HbA1C, glycosylated hemoglobin; HOMA-IR, HOMA-Insulin Resistance.

Table S7 Stratified associations between maternal glucose metabolic markers and offspring total difficulties scores by child sex

	**Total Difficulties Score, *OR* (95%*CI*)**	***P***
**GDM**		
boy	3.12 (1.06, 9.25)	0.284
girl	1.19 (0.30, 4.84)	
**FPG**		
boy	**1.75 (1.11, 2.78)***	0.347
girl	1.16 (0.56, 2.41)	
**OGTT-1h glucose**		
boy	1.46 (0.88, 2.42)	0.925
girl	1.41 (0.78, 2.54)	
**OGTT-2h glucose**		
boy	**1.79 (1.12, 2.88)***	0.129
girl	0.96 (0.50, 1.85)	
**HbA1c**		
boy	1.46 (0.86, 2.47)	0.305
girl	0.96 (0.53, 1.75)	
**Insulin**		
boy	1.14 (0.68, 1.92)	**0.045**
girl	**2.87 (1.37, 5.99)***	
**HOMA-IR**		
boy	1.37 (0.81, 2.34)	0.151
girl	**2.65 (1.29, 5.44)***	

*$P<0.05$, Statistically significant ($P<0.05$) results were in bold.

a The main model was adjusted for maternal age, maternal educational level, pregnancy BMI, gestational weight gain, family history of diabetes, maternal smoking, physical activity, dietary energy intake, child's sex, mode of delivery, weeks of gestation, birthweight, exclusive breastfeeding complementary feeding age.
